# Patients’ and Providers’ Perceptions of the Implementation of a Stepped Care Cognitive-Behavioral Therapy for Insomnia in Routine Cancer Care: A Qualitative Post-Implementation Study

**DOI:** 10.3390/curroncol33100588

**Published:** 2026-09-30

**Authors:** Lauriane Giguère, Émilie Godin, Claudia Mc Brearty, Aude Caplette-Gingras, Marie-Pierre Gagnon, Lynda Bélanger, Charles M. Morin, Josée Savard

**Affiliations:** 1 School of Psychology, University of Ottawa, 75 Laurier Ave E, Ottawa, ON K1N 6N5, Canada; lgigu023@uottawa.ca; 2 Université Laval Cancer Research Center, 11 Côte du Palais, Québec, QC G1R 2J6, Canada; emilie.godin@crchudequebec.ulaval.ca (É.G.); claudia.mc-brearty.1@ulaval.ca (C.M.B.); aude.caplette-gingras.1@ulaval.ca (A.C.-G.); 3 School of Psychology, Université Laval, 2325 Allée des Bibliothèques, Québec, QC G1V 0A6, Canada; cmorin@psy.ulaval.ca; 4 CHU de Québec-Université Laval Research Center, 1401 18e Rue, Québec, QC G1J 1Z4, Canada; 5 Centre des Maladies du Sein, CHU de Québec-Université Laval, 1050 ch Ste-Foy, Québec, QC G1S 4L8, Canada; 6 School of Nursing, Université Laval, 1050 Avenue de la Médecine, Québec, QC G1V 0A6, Canada; marie-pierre.gagnon@fsi.ulaval.ca; 7 CHU de Québec-Université Laval, 10 Rue de l’Espinay, Québec, QC G1L 3L5, Canada; lynda.belanger@chudequebec.ca; 8 CERVO Brain Research Center, 2301 Avenue d’Estimauville, Québec, QC G1E 1T2, Canada

**Keywords:** cancer survivors, cognitive-behavioral therapy, implementation science, insomnia, survivorship care, stepped care

## Abstract

Insomnia is one of the most commonly reported challenges among cancer patients and survivors. Cognitive-behavioral therapy for insomnia (CBT-I) is an effective treatment but remains underused in routine cancer care. A stepped-care CBT-I program was developed to address this gap. This study explores facilitators and barriers following the implementation of this program in four Québec cancer centers. Patients and providers participated in two individual interviews and seven focus groups. Interviews were guided by the Consolidated Framework for Implementation Research (CFIR). Data were transcribed and coded in NVivo using the CFIR codebook. A hybrid deductive–inductive thematic analysis was conducted. Codes were interpreted using Proctor’s Implementation Outcomes Framework. A total of 14 patients and 18 providers were interviewed. Facilitators and barriers were divided into four key themes: (1) endorsement from trusted professionals; (2) fit with workflows and existing routines; (3) perceived burden for both patients and providers; and (4) readiness and motivation of both patients and providers. This analysis supports the acceptability, adoption, appropriateness, feasibility, and perceived sustainability of a stepped-care CBT-I in routine cancer care and suggests specific implementation strategies to optimize its implementation. Implementation of stepped-care CBT-I in routine cancer care has the potential to improve access to evidence-based treatments for insomnia and make it a standard component of survivorship care.

## Simple Summary

Insomnia is a commonly reported challenge for cancer patients and survivors. While cognitive-behavioral therapy is useful to treat insomnia, it is not always available in routine cancer care. Our team developed a step-by-step program to address this gap. We tested this program in four Québec cancer centers. We then interviewed a small group of cancer patients who volunteered to take part in this study, as well as healthcare providers, to understand what facilitated or hindered putting this program into practice. Key factors included support from trusted professionals, fit with existing routines, the effort required to use it, and the motivation of all those involved. Overall, this study suggested that our program can be successfully integrated, allowing better access to insomnia care for patients and survivors.

## 1. Introduction

Between 30 and 60% of cancer patients are affected by insomnia [1,2,3]. Timely treatment for insomnia is crucial as 29–64% of patients may develop chronic insomnia if no intervention is offered [3]. Considering the importance of sleep on one’s psychological and physical functioning [4], particularly when living with cancer, untreated chronic insomnia bears a significant toll both on the individual and the healthcare system. Indeed, chronic insomnia is associated with an array of negative consequences including greater fatigue, psychological distress, cognitive impairments, diminished quality of life and daytime functioning, higher risk of infection during chemotherapy, and a greater utilization of health care services [5,6,7]. In addition, epidemiological studies have consistently established insomnia as a significant risk factor for the subsequent development of depressive and anxious disorders [5,8,9], including in cancer survivors [10,11].

As in other clinical contexts, insomnia is frequently overlooked in cancer care. Sleep is not routinely screened by cancer care providers [12,13,14]. Furthermore, patients often prioritize sharing other concerns when meeting with their oncology team [12,13]. In addition, many patients are ambivalent towards using a sleep medication, further deterring them from discussing insomnia. Yet, sleep medication is, by far, the most frequently prescribed and routinely used treatment for insomnia, with many patients exceeding the maximum daily usage of 4 to 6 weeks [15,16] recommended by the National Institute of Health and sleep experts’ associations [17,18].

Cognitive-behavioral therapy for insomnia (CBT-I) is the treatment of choice for chronic insomnia both in the general population and among cancer patients [19,20]. Its efficacy in treating cancer-related insomnia has been supported by numerous clinical trials [21,22,23] showing not only short- and long-term improvements in insomnia severity and sleep parameters (e.g., sleep efficiency, number and duration of awakenings), but also parallel reductions in levels of anxiety, fatigue, depression, and subjective cognitive impairments, as well as quality of life improvements. In addition, CBT-I is more efficacious than other interventions such as sleep hygiene education, acupuncture, mindfulness-based stress reduction interventions, hypnotic medications, and waitlist controls [24,25,26].

However, access to CBT-I remains limited. Several factors may explain this gap, such as the lack of expertise in oncology teams to administer it, the time and costs involved in offering in-person CBT-I, as well as shortages in psychosocial oncology resources and prioritization of more severe problems such as depression and anxiety. However, there is recent evidence showing that successful early treatment of insomnia is an effective intervention to prevent the development of psychological disorders that are more severe, more incapacitating, and more challenging and costly to treat than insomnia, such as depressive disorders [27]. For instance, a recent randomized controlled trial (RCT) found that CBT-I prevented the incidence and recurrence of major depression in elderly individuals (60 years and older) with insomnia [27].

It is often believed that insomnia will fade away by itself with a successful treatment of depression or anxiety. However, studies with non-medically ill individuals have shown that residual insomnia is common following such treatment, with rates varying from 30 to 50% over a 3–5-year follow-up [28,29]. We recently found similar results in women with metastatic breast cancer [30]. Although cognitive therapy for depression was overall effective in reducing depressive symptoms, residual insomnia was observed in 21% of the participants at a 6-month follow-up [30]. This indicates that a specific insomnia treatment is needed for a substantial proportion of patients.

Our research team has played a significant role in the development and assessment of low-intensity treatment formats to make CBT-I more accessible to patients with cancer [31,32]. More recently, we developed a stepped-care CBT-I intervention beginning with an unguided web-based program (called Insomnet), followed by up to three booster sessions with a psychologist if the patient has residual insomnia following the completion of Insomnet. Our RCT showed that effects of our stepped-care CBT-I, stratified based on baseline insomnia severity, were not significantly inferior to standard CBT-I composed of 6 weekly treatment sessions [31]. From then on, we concluded that efforts needed to be shifted to implementing our stepped-care CBT-I in routine cancer care.

We then undertook a 3-phase implementation study, called IMPACT (Insomnia in Patients with Cancer—Personalized Treatment) program, which was aimed at assessing the feasibility and efficacy of implementing our stepped-care CBT-I in four cancer centers. A pre-implementation qualitative study was conducted among 43 clinicians and 6 administrators to identify the barriers to and facilitators associated with implementing this intervention in their clinical settings [32]. Results confirmed the need to better screen and treat insomnia in oncology settings and identified facilitators and barriers that were addressed in the subsequent implementation phase (Phase 2; Savard et al., submitted). The current manuscript presents results of Phase 3 of this larger study, a post-implementation qualitative investigation, which was aimed at evaluating patients’ and providers’ (i.e., individuals involved in the provision of healthcare directly or through administrative tasks) [33] perceptions about the barriers and facilitators of implementing a stepped-care CBT-I program in routine cancer care as well as the program’s acceptability, adoption, appropriateness, feasibility, and potential sustainability.

## 2. Materials and Methods

### 2.1. Participants

The IMPACT program was implemented in four hospitals providing cancer care in the area of Québec City, QC, Canada: Centre intégré de cancérologie and Hôpital du St-Sacrement, both of which are part of the CHU de Québec-Université Laval; Centre régional intégré de cancérologie de l’Hôtel-Dieu de Lévis; and Institut universitaire de cardiologie et de pneumologie de Québec. At the time of Phase 1, five hospitals were involved in the study, but the cancer services of two of them merged (L’Hôtel-Dieu de Québec and Hôpital de l’Enfant-Jésus) during the course of the project to form the Centre intégré de cancérologie of the CHU de Québec-Université Laval. Focus groups were conducted with two types of participants: (1) patients who participated in the program; and (2) providers who played a role in implementing IMPACT. Individual interviews could be offered as alternatives to focus groups.

Patients were selected among those who had provided their consent to be contacted for further participation after completing the IMPACT program. To be retained for this study by the referral algorithm, participants were required to: (1) be healthy enough to participate (i.e., their medical condition(s) should not interfere with attendance and participation in a one-hour discussion in terms of pain, energy, or concentration levels); (2) have a sufficient level of French; and (3) wish to share an opinion on the importance of treating sleep disturbances in cancer patients and on the implementation of stepped-care CBT-I in routine care. Efforts were made to recruit a diverse group of patients in terms of age, sex, living area, main cancer site, treatment site, and completion status of Insomnet (completed, uncompleted, completed with booster sessions).

Providers were selected by a point person in each hospital who knew the project well. An emphasis was placed on possible variety of points of view and opinions (i.e., early-career and more experienced providers), professions (e.g., nurse, psychologist, social worker, medical doctor), and role in the implementation process. For both patients and providers, all interested participants were invited to indicate their availability. The date and time when most interested participants could be present was chosen. Therefore, those who did not participate were either no longer interested or not available at that time.

For both groups, data collection ended at a pragmatic endpoint: when all available and interested participants were interviewed. The information and consent form was sent by email, and written consent was provided before the interview.

### 2.2. Procedure

#### 2.2.1. Implementation of Stepped-Care CBT-I in Routine Care

The study used a hybrid effectiveness-implementation and a stepped-wedge cluster non-randomized design to implement the stepped-care CBT-I at various time intervals across cancer centers. Based on results of the pre-implementation qualitative study (Phase 1) [32], a general implementation plan was designed, which was slightly adapted to better reflect the functioning mode of each hospital. All first-line cancer care providers (e.g., nurses, oncologists, radio-oncology technologists, pharmacists) at the four hospitals were informed every 3–4 months about the implementation project, uptake and completion rates, and reminded of how to refer patients to stepped-care CBT-I as part of routine care. Specifically, they were made aware of the significance of cancer-related insomnia and its negative impact, the limitations of pharmacotherapy, the empirical evidence supporting the efficacy of CBT-I and stepped-care CBT-I, and the importance of better screening and treating it. Insomnia screening was mainly done using the sleep item of the Edmonton Symptom Assessment System-Revised (ESAS-R-sleep) [14,34] as part of a systematic screening of psychological distress previously implemented in some departments (e.g., chemotherapy and radio-oncology units). For departments not using this screening procedure or at times when systematic screening was not planned, providers could rely on other measurement tools they were using or a patient’s subjective sleep complaint and clinical judgment. For the ESAS-R-sleep item, a score of 4 or greater was used to determine the presence of insomnia [14,35]. This was consistent with other cut-off scores used for screening other types of difficulties (e.g., depression, anxiety) in these settings [36]. This is greater than the cut-off score of 2 suggested by our earlier study contrasting the ESAS-R-sleep item with the Insomnia Severity Index (ISI) [14] to maximize sensitivity, but was chosen to avoid overburdening psychosocial oncology services [35]. Specifically, this score was associated with a sensitivity of 59.4% and a specificity of 96.0%.

Providers were instructed to give a leaflet explaining the stepped-care CBT-I and how to access the web-based program (Insomnet; Step 1) to patients having insomnia. Based on a referral algorithm, patients who had more severe psychological difficulties (e.g., depressive or anxiety disorder), severe cognitive impairments, low literacy, or no access to the Internet were referred to each hospital’s psychosocial oncology service. At registration, patients had the opportunity to provide their consent to complete a pre- and post-intervention battery of self-report scales and to be contacted for other studies by our research team. As the stepped-care CBT-I was offered within routine care, patients were informed that completion of these questionnaires was optional. Regardless of this decision, all participants had to complete the ISI before and after Insomnet and provide daily sleep diary data to document intervention effectiveness (not reported here). Patients with residual insomnia symptoms after completing this first step, as defined by a score of 8 or greater on the ISI, were offered 1–3 booster sessions with a clinical psychologist from the hospital’s psychosocial oncology team (Step 2). This referral procedure was automated so that both the patient and provider responsible for the program in each setting received an e-mail informing them of patients’ eligibility for booster sessions. The patient, if interested, was then placed on the waiting list to receive booster sessions.

#### 2.2.2. Interviews (Supplementary Material S1) [37]

Focus groups were conducted at the respective hospital where patients were treated when they were referred to the IMPACT program between October 2023 and June 2024. They were conducted by a facilitator from a professional survey company (SOM) in order to reduce social desirability bias. The facilitator had 13 years of experience at this company at the time of this study. They had no prior relationship with the participants. A semi-structured interview guide was used (see Appendix A) based on the initial version of the CFIR model (i.e., before its update) [38]. Before focus groups, only providers were asked to complete a short 4-item study-specific sustainability questionnaire about whether the program should be maintained, its perceived sustainability, relevance, and usefulness. They provided their responses on a 5-point scale (1 = completely disagree to 5 = completely agree). These questions were not provided to patients given that they related to internal institutional issues. Instead, patients were questioned on these issues during their interviews or focus groups, giving the opportunity for additional explanations which would have been impossible in a questionnaire form.

### 2.3. Data Analysis

Interviews were audio-recorded and transcribed verbatim by an undergraduate psychology student. Verbatim transcriptions were imported into NVivo 11.0 [39], and a hybrid inductive-deductive codebook thematic analysis was performed [40]. The deductive thematic analysis started with the five CFIR domains (before the update) that are associated with effective implementation (i.e., *Intervention Characteristic*, *Outer Setting*, *Inner Setting*, *Characteristics of Individuals*, and *Process*). Additional codes were added to the initial codification tree as needed during the analysis. All interviews were analyzed independently by two raters (EG and a graduate student in psychology) with significant experience in qualitative analyses. They compared their coding and discussed disagreements until they reached consensus on a common codification tree. Each code was operationalized. A review of the codes was done by LG to match the updated CFIR coding classification (CFIR framework 2.0) [41,42]. LG used the Proctor’s Implementation Outcomes Framework to contextualize codes and implementation success [43]. These include support for acceptability (i.e., satisfaction with innovation as opposed to other options), adoption (i.e., uptake of the innovation), appropriateness (i.e., perceived fit and compatibility of the innovation with the setting), feasibility (i.e., potential for the innovation to be successfully used within the setting), and potential sustainability (i.e., ability to maintain the innovation and its viability in the long term). Then, she grouped these implementation outcomes into cross-cutting themes which were validated by EG and JS. Of note, LG was not involved in the development or implementation of the IMPACT program. Relatedly, all researchers involved in coding (LG, EG, CMB, and JS) have backgrounds in clinical psychology and psycho-oncology. Some of us also have personal experiences with cancer and work in cancer centers. Given this shared background, we may have been more attuned to clinical and psychological dimensions of the data as opposed to organizational or systems-level dimensions. Notably, LG has also been trained in program evaluation. In grouping findings into themes and writing this manuscript, LG actively reflected on her own perspectives, biases, and assumptions. Of note, all study results were initially handled in the original language (French), and participants’ quotes were translated for the purpose of publication only, by a professional research translator who has been working with this team for several years. The quotes provided in this manuscript were also verified by LG and JS, who are both fully bilingual.

## 3. Results

A total of 29 patients and 45 providers (i.e., n = 15 managers and n = 30 clinicians) gave their consent to be contacted for the study. Fourteen patients and 18 providers (i.e., n = 6 managers and n = 12 clinicians) took part in focus groups. There were a total of three focus groups and two individual interviews with patients and four provider focus groups. Individual interviews were offered to one participant who was uncomfortable with the group setting and to another who had expressed interest too late. Focus group duration ranged from 56 to 96 min. Individual interviews lasted 30 to 36 min.

Among the participating patients, three did not complete the program for undisclosed reasons, and five received booster sessions due to their ISI indicating residual insomnia. Table 1 and Table 2 present patients’ and providers’ demographic and clinical characteristics, as well as ISI scores before the interviews or focus groups and providers’ opinions on whether the program should be maintained, its perceived sustainability, relevance, and usefulness. Of note, one patient did not provide their demographic characteristics but was counted nonetheless. Patients’ mean ISI score before the interviews and focus groups indicated a clinical level of insomnia, with individual levels widely ranging from not clinically significant to clinically significant (severe; mean = 9.1, range = 1–24). Providers’ scores indicated that they were in strong agreement with the program being maintained (mean = 4.24), its perceived sustainability (mean = 4.35), its relevance (mean = 4.24), and its usefulness (mean = 4.24).

### 3.1. Thematic Analysis

**Figure 1:**
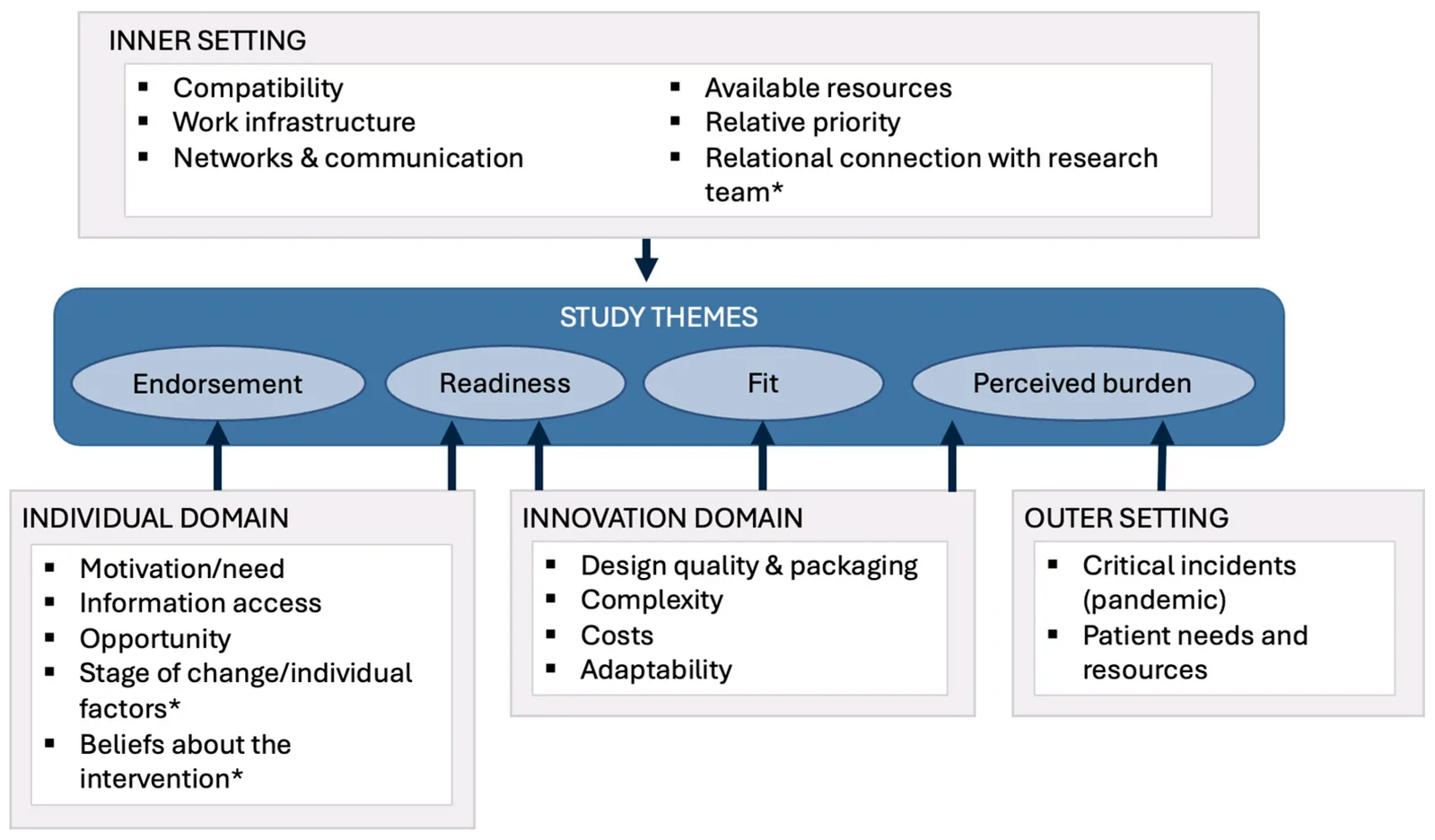
The relationship between CFIR and inductive themes and cross-cutting themes is depicted in this figure. Inductive themes are identified by an asterisk (*).

Four cross-cutting themes were identified as central to a successful implementation: (1) endorsement as a pathway to credibility; (2) readiness and motivation influencing adoption; (3) perceived burden influencing adoption, feasibility, and potential sustainability; and (4) fit with workflows as a determinant of potential sustainability. These mechanisms supported acceptability, adoption, appropriateness, feasibility, and perceived sustainability. Figure 1 demonstrates the link between these themes and CFIR domains. Table 3 presents participants’ quotations for each theme.

#### 3.1.1. Endorsement as a Pathway to Credibility

A dominant factor across all interviews was provider endorsement as a central mechanism for the credibility of the intervention and for patient engagement. For patients, providers who appeared unfamiliar with the program hindered their participation. Repeated and positive discussions between patients and providers about the intervention increased the likelihood of it being perceived as important and useful, thus increasing patient engagement and further cementing the program’s place in their hospital setting.

Providers reported that a lack of information (e.g., not being able to talk properly about the program due to a lack of knowledge and understanding) posed a barrier to their endorsement of the program. Nonetheless, they believed that the program was an excellent addition to their services. They believed in its added value and attempted to communicate this to patients. This was crucial considering the weight participants placed on their providers’ opinion of the program.

Overall, it was evident that providers’ beliefs acted as a gatekeeping mechanism to patient engagement. Patients’ perception of their providers’ beliefs toward the intervention was key to credibility, acceptability, and adoption of the program. However, providers seemed unaware of the importance their patients attributed to their beliefs regarding the intervention’s efficacy, added value, and fit for their patients. Clearly communicating providers’ positive beliefs about the program to patients may be a crucial tool to its implementation and potential sustainability.

#### 3.1.2. Readiness and Motivation Leading to Acceptability and Adoption

Crucial to adoption of the program was readiness and motivation for both patients and providers. Introduction to the program was highly dependent on patients’ or provider-perceived stage of change in terms of intrinsic motivation, commitment to treatment, perseverance, and opportunity. Perceived stage of change was influenced by providers’ own assumptions regarding a patient’s readiness, motivation, and opportunity. Due to these perceptions, some providers occasionally selected specific patients as “ideal” patients, inadvertently excluding patients with no contraindication (e.g., older age, perceived likelihood of adherence). This may have facilitated uptake as providers ensured adequate fit for patients, but this may have limited accessibility.

Providers’ adoption of the program and motivation to promote it was also dependent on their own stage of change, which was influenced by opportunity and readiness. In other words, providers reported difficulties integrating the promotion of the program into their routine or easily falling back into old habits. These were, in turn, influenced by an overflowing schedule and reportedly not having a concise and effective way of promoting the program, despite the research team providing extensive material to that end (e.g., examples of sentences to use to present the program). This appeared to have been a result of a lack of time or resources. Interestingly, a lack of uptake from patients was also an important moderator of providers’ motivation to continue offering the program.

Finally, content and packaging were also important to patients’ motivation. Accessibility (i.e., free access, using videos), ease of use (i.e., health literacy), adequate pace (i.e., one module per week) and interactivity (i.e., interactive automatic messages by the platform) increased engagement and facilitated uptake and adherence. This also limited providers’ burden by decreasing patients’ need for support and therefore increasing their motivation to promote the program.

#### 3.1.3. Perceived Burden Influencing Adoption, Feasibility and Potential Sustainability

Perceived burden looked different for patients and providers, but both remained crucial to referral and adherence. On patients’ side, the program’s complexity (high level of engagement required, counterintuitive techniques), pace, and free access influenced the perceived burden of the intervention. Interestingly, this was facilitated by the COVID-19 pandemic restrictions during implementation, which offered time and energy to focus on this program.

For providers, perceived patient burden was the most important factor. Consideration for patient time and effort held some providers back from actively promoting the intervention, citing potential attrition because of resource-heavy demands. Also important was the support provided to providers by the research team through the provision of uptake and completion statistics, informational materials, and technical or organizational resources to help providers better promote the intervention. Indeed, the research team’s presence to answer questions and problem-solve challenges that arose was a major facilitator.

Beyond the research team’s presence, providers naturally relied on each other to implement the program by sharing accountability and responsibilities across team members. By encouraging communication and initiation rather than completeness, individual expectations were lowered, and continuity of care was ensured without relying on a single provider. In other words, reliance on the circle of care reduced the individual burden of integrating this program into their daily workflows.

#### 3.1.4. Fit with Workflows for Feasibility and Potential Sustainability

Overall, patients identified a good fit with workflows and systems as facilitators that increased feasibility and potential sustainability. Some patients identified issues of equal accessibility through cancer treatment modalities. A few participants reported that only providers involved in chemotherapy treatments promoted IMPACT, while providers involved in radiotherapy and surgery did not since they had limited time with patients. Timing of treatment (e.g., evenings, weekends) also impacted participants’ frequency of contact with providers and therefore limited opportunities to promote the program. Standardizing the offer across cancer and treatment types was reported to be crucial to equity of care. The structure and topic of appointments also determined the subjects discussed (e.g., test results, physical symptoms, medication adjustments). For example, clinical appointments between patients and oncologists may be short and centered on test results, medication adjustments, or other issues directly related to cancer treatment rather than on psychosocial survivorship care, where insomnia may not be a priority. Even more important, some patients did not feel that anxiety-inducing appointments (i.e., where they received test results) were conducive to discussing insomnia. Consequently, patients reported that having documents provided to them or resources they could consult after their appointments facilitated the processing of information and subsequent enrollment in the program.

Providers had different concerns. Issues of overburdened workflows, high staff turnover or short-staffing, and opportunity for adequate follow-up highlighted the healthcare system’s structural and systemic barriers. Despite this, implementation was facilitated by the purposeful design of the intervention (i.e., self-administered and automatic referrals that required minimal provider involvement), making it amenable to low levels of provider support. Of note, providers at one study site were initially supported by a dedicated professional who systematically contacted referred patients over the phone to explain the program and answer patients’ questions, which supported their uptake and engagement. While this facilitated program registration and retention, providers acknowledged the limited generalizability and maintenance of this practice. As a matter of fact, the follow-up phone contacts were eventually stopped as this professional was no longer available to do these. Further, the presence of the research team throughout implementation was cited as a critical facilitator to timely modifications and adaptations of the program to fit with workflows. The research team also supported implementation consistency through regular reminders for providers.

Overall, implementation may require structural and systemic adjustments to support equal access and feasibility and minimize strain on resources, particularly as the program develops and grows. Notably, early implementation success for one institution was partially dependent on dedicated support and presence of the research team, raising some questions about growth potential once external scaffolding is withdrawn. However, providers’ flexibility and the intervention’s adaptability when faced with challenges were promising for long-term implementation.

#### 3.1.5. Additional Considerations

Participants made several suggestions to improve the content, format, and support offered during the intervention. Regarding content, some of the suggestions included having a lay summary or reminder document at the end to summarize the program and linking other helpful resources to the platform, such as resources for anxiety or depression. In terms of format, some participants suggested having a paper version for those who have lower technology literacy, while others suggested an application for their phones to improve accessibility. Finally, many participants wished for a dedicated follow-up meeting during the intervention to check in with health care professionals about progress, questions, or concerns.

Similar to patients, providers suggested ways to improve the content and support offered to providers and patients. For content, they suggested creating videos to familiarize patients and providers with the platform and with stepped-care CBT-I. They also suggested a toolbox for providers containing promotional material, motivational and enrolment strategies, and available additional support. It is important to note that researchers did provide an important number of these resources (e.g., promotional material including pins, mugs, pens with the study logo, posters, proposed verbatim to use to present the program, videos and pamphlets) that appeared to have been poorly used. Similarly, some providers suggested providing a support telephone line available during the intervention for patients and providers, even though these resources had been provided but also went unused.

## 4. Discussion

The goal of this study was to explore patients’ and providers’ opinions on the facilitators and barriers of implementing stepped-care CBT-I in their hospital settings. To our knowledge, this is the first study to assess implementation of an insomnia stepped-care program and, among the few [44,45,46,47], to implement a psychological intervention in routine cancer care. Overall, both patients and providers favored maintaining long-term implementation of stepped-care CBT-I in their hospital settings. Patients and providers identified barriers and facilitators that were consistent with the CFIR framework. Collectively, the themes suggested that implementation success was driven by providers’ endorsement, compatibility with workflows, minimized burden on both patients and providers, as well as individual motivation influenced by stage of change. While the well-established intervention’s efficacy provided a strong rationale for initiating implementation, these mechanisms operated across both groups to support acceptability, adoption, appropriateness, feasibility, and perceived sustainability.

By using the CFIR, we were able to generate a structured understanding of barriers and facilitators and systematically identify contextual factors influencing implementation. Inner setting factors were consistently reported across all themes, cementing their importance as the backbone for the implementation of this intervention. This led us to believe that adequate fit with the inner setting must be given significant attention to successfully implement this intervention. Individual factors were also crucial early on for endorsement and readiness, while innovation domain factors and outer setting factors became increasingly relevant at the latter end of the process with regard to potential sustainability (i.e., readiness, perceived burden and fit; see Figure 1). This is consistent with previous implementation studies suggesting that champions are crucial determinants of change for both proximal (i.e., readiness for change, adoption) and distal (i.e., sustainability) implementation outcomes [48,49].

Some of the barriers and facilitators mentioned by each group were closely related to what participants had mentioned in pre-implementation interviews [32]. Prior to implementation, providers had identified resistance to change as a potential barrier to implementation, which was also identified post-implementation. While resistance to change was not explicitly cited in this study, time constraints were frequently identified as potential barriers. This was apparent in providers’ lack of familiarity with the resources provided to them and the program itself; they were encouraged to consult them, but few did. Nonetheless, the impact of time constraints may have been mitigated by adequate support from the research or healthcare team, which minimized resistance and ambivalence. Based on suggestions from the pre-implementation qualitative study, the research team also prioritized brevity and made significant efforts to reduce the time necessary to promote or support the program (e.g., referral flyer, automated referral system to booster sessions). In addition, offering frequent reminders and training to employees to mitigate high turnaround rates was described pre- and post-implementation. Although meetings with clinical teams took place regularly, this was not always sufficient, suggesting that additional strategies should be adopted. Overall, integration appeared feasible and may be sustainable when sufficient considerations to mitigate inner setting and individual domain barriers are taken into account. For example, due to cancer treatments’ impacts on patients, the timing of the intervention was crucial. Furthermore, ensuring continuity of care (i.e., ongoing provider-patient relationship, shared knowledge across providers) for both patients and providers was considered important to their motivation. Given the limited resources allocated to psychosocial services in a public hospital setting, which may interfere with the implementation of such interventions, providers in this study suggested the involvement of a third party (e.g., community-based organizations) that could spearhead and manage this program alongside the healthcare providers. They observed that coordination across hospitals and care settings could be more effectively facilitated by a third-party organization rather than by centralizing the intervention in one hospital. This may help reduce the burden on the healthcare system and promote the program’s potential sustainability, as the research team did during this study. Similarly, pre- and post-implementation, patients reported concerns about competing priorities during appointments, which may steer conversations away from insomnia concerns. Other implementation efforts of psychological interventions such as fear of cancer recurrence group therapies (e.g., FORT, SWORD, ConquerFear) [44,50,51] were also associated with similar observations as the ones highlighted in this study, showing that these themes may transpire across intervention types and implementation efforts.

Relatedly, there has been a shift toward principle-based stepped-care approaches which center around values such as client readiness and choice along with operational flexibility and responsiveness of the system [52]. Consistent with this approach, our results showed that patients and providers needed an adaptive approach that avoided fragmentation, poor service coordination, and limited accessibility. They required a system that integrated assessment of needs, encouraged self-awareness, and supported patient agency while also limiting provider burden. The referral to Insomnet (first step) through a leaflet and the subsequent automated referral system to move patients from the first to the second step of the program responded well to these needs and minimized fragmentation, also ensuring adequate follow-up and follow-through. In addition, the culture nurtured by the research team promoted collaboration and teamwork, supporting the providers through their consistent, reliable, and expert presence.

Finally, the findings of this study are consistent with previous research identifying key facilitators and barriers to the implementation of internet-based psychological interventions. Berardi and colleagues’ [53] systematic review highlights the importance of both individual and clinician-level factors in influencing implementation. They cited negative perceptions, perceived burden, and limited resources as key barriers, and person-centered approaches, training and support for clinicians, collaboration among professionals, and adequate human resources as key facilitators. These were replicated in our study’s overarching themes, showing that successful implementation requires coordination at the individual and systemic level. In another study, attitudes and beliefs toward digital interventions, as well as perceived time constraints, burden, and availability of resources, played an important role in shaping patients’ motivation to engage with these interventions [54]. Similarly, at the healthcare provider level, providers’ attitudes and beliefs, competing time demands, resource availability, integration within mental healthcare services, and characteristics of the technology itself (i.e., design, usability, and content) have been identified as important determinants of successful implementation. In addition, healthcare providers’ knowledge, education, and training related to digital interventions may further facilitate their adoption in clinical practice. In our study, although providers had the opportunity to look at Insomnet, only a few did. Our findings suggest that better knowledge of the intervention and its online platform would be useful for sustained implementation.

### 4.1. Limitations

Some limitations need to be acknowledged. First, the limited sample size, number of participating sites, and the qualitative nature of this study reduce the results’ generalizability to other hospitals outside of the province of Québec. Second, the sample was mostly female, which may have affected our findings. Third, the setting in which this study took place (i.e., universal healthcare) may limit its results’ applicability to other settings with differing healthcare models (e.g., the United States). Fourth, voluntary participation following the intervention, as well as the reference algorithm that was used by cancer care providers to determine the appropriateness of the program for patients, may have introduced a self-selection bias, as patients with certain characteristics (e.g., technology literacy) who appreciated their experience may have been more likely to participate in this qualitative study. The reference algorithm also introduced boundaries to the evaluation of accessibility, considering the imposed barriers on individuals with limited digital access, lower literacy, language needs, disabilities, or limited time and energy. Of note, the cut-off score chosen on the ESAS-R-Sleep also impacted the selection and referral of participants to the intervention. That is, the cut-off chosen implied a 40% false-negative rate, increasing the likelihood of missing individuals with marginally above-threshold clinical insomnia. This may have skewed the distribution and representativeness of patients targeted by the intervention to favor those with severe insomnia. However, while this limits our ability to support this intervention’s implementation with moderate to marginally above-threshold cases, it is indicative of the potential for this intervention for patients with more severe insomnia. Fifth, it is possible that there may have been additional self-selection bias by having site point persons recommend providers to participate in focus groups who could be more enthusiastic about the IMPACT program and its integration in routine care.

### 4.2. Clinical Implications and Future Directions

These study results suggest that our stepped-care insomnia intervention has its place alongside other supportive care provided to patients in routine cancer care. However, the importance of fit with resources as well as responsiveness to changes in workflows and workloads must be considered for successful implementation. Individual fit with the intervention (i.e., motivation/need, stage of change, opportunity) was also paramount in ensuring acceptability and adherence. These results can help inform future implementation efforts of evidence-based psychological interventions into real-world cancer care.

## 5. Conclusions

Overall, there is a great interest from both patients and providers in implementing stepped-care CBT-I into oncology settings. Of note, a stepped-care approach allows flexibility and adapts to patients’ needs. It may improve access in terms of financial means, time resources, and energy levels as opposed to resource-intensive, face-to-face CBT-I, despite barriers for individuals with limited digital access, lower literacy, language needs, disabilities, and limited time and energy. It offers an alternative to the pharmacological treatments typically offered and that have many limitations. Providers did not feel that it strained their resources but did raise concerns about the absence of the research team to support its long-term integration, which should continue to be monitored and proactively addressed. Considering the prevalence of sleep issues as a source of unmet needs for cancer patients and survivors, as well as its impact on their health-related quality of life [4], providing an evidence-based, effective, and accessible intervention to cancer survivors is critical to supportive care in the context of routine cancer care. This study suggests that implementation is possible, potentially feasible, and acceptable.

## Figures and Tables

**Table 1:** Patients’ demographic and clinical characteristics (*N* = 14).

	Mean (Range)
Age	53.6 (39–81)
Sex (*N* = 13)	
Female	12 (92.3%)
Male	1 (7.7%)
Cancer type (*N* = 14)	
Breast cancer	11 (78.6%)
Colorectal cancer	1 (7.1%)
Prostate cancer	1 (7.1%)
Oropharyngeal cancer	1 (7.1%)
Marital status (*N* = 13)	
Married/cohabitating	8 (61.5%)
Unpartnered	5 (38.5%)
Education (*N* = 13)	
High school	2 (15.4%)
College	6 (46.1%)
University	5 (38.5%)
Occupation (*N* = 13)	
Full-time work	0 (0%)
Part-time work	2 (15.4%)
Sick leave	5 (38.5%)
Retired	4 (30.8%)
Other	2 (15.4%)
Family income (CAD) (*N* = 13)	
≤$40,000	1 (7.7%)
$40,001–$60,000	3 (23.1%)
$60,001–$80,000	2 (15.4%)
$80,001–$100,000	2 (15.4%)
$100,001–$120,000	1 (7.7%)
≥$120,001	3 (23.1%)
Prefer not to answer	1 (7.7%)
Cancer treatments ^1^	
Surgery	6 (46.2%)
Radiation therapy	7 (53.8%)
Chemotherapy	6 (46.2%)
Hormone therapy	5 (38.5%)
Unknown	2 (15.4%)
Program completed (*N* = 14)	
Yes	11 (78.6%)
No	3 (21.4%)
Booster sessions received (*N* = 11)	
Yes	5 (45.5%)
No	6 (54.5%)
ISI total score	Mean (Range)
	9.1 (1–24)

^1^ Some categories may not add up to 100% as patients identified with more than one option (i.e., treatment type).

**Table 2:** Providers’ demographic and professional characteristics (*N* = 18).

	Mean (Range)
Age	39.2 (28–55)
	*N* (%)
Sex (*N* = 18)	
Female	17 (94.4%)
Male	1 (5.6%)
Marital status (*N* = 18)	
Married/cohabitating	16 (88.9%)
Unpartnered	2 (11.1%)
Education (*N* = 18)	
University	18 (100%)
Hospital (*N* = 18)	
Hôpital du Saint-Sacrement	6 (33.3%)
IUCPQ	7 (38.9%)
Hôtel-Dieu de Lévis	3 (16.7%)
L’Hôtel-Dieu de Québec/CIC-CHU	2 (11.1%)
Years of experience at their hospital	Mean (Range)
	9.75 (1.5–32)
Years of experience in the healthcare system	Mean (Range)
	14 (5–32)
Years of experience in oncology	Mean (Range)
	7.75 (0–32)
Job position (*N* = 17)	
Social work	2 (11.7%)
Psychology	4 (23.5%)
Nurse	6 (35.3%)
Pharmacist	2 (11.7%)
Nutritionist	1 (5.8%)
Medical doctor	1 (5.6%)
Administrator	1 (5.6%)
Belief IMPACT should be maintained	Mean (Range)
	4.24 (3–5)
Perceived sustainability of IMPACT	Mean (Range)
	4.35 (4–5)
Relevance of service	Mean (Range)
	4.24 (2–5)
Useful for patients	Mean (Range)
	4.24 (3–5)

**Table 3:** Quotes per theme.

Theme	Participant Type	Quote
Endorsement	Patient	“As I say, both of them talked to me about it. Already at that point, I thought this was really good, and I even think my nurse navigator had also mentioned it to me. So, you see, that’s two or three people who had talked to me about it.” (Individual interview I001, Hospital 02) “Maybe just raise awareness among the nurses a little more—they’re maybe not all aware to the same extent. […] Because the nurses are the people we confide in a lot. We hardly ever see the doctors, but we see the nurses—we see them for blood tests, for treatments, we call them by their first name. So I think that better information for nurses could help.” (Participant 04, Hospital 01)
	Provider	“So, I think a minor issue is that the doctor who refers someone to the IMPACT program can’t necessarily provide as much information as, say, someone like me who’s familiar with the program material. So, of course, I think this can sometimes maybe slow down the referral process, when you want to give a patient a tool, but you’re not necessarily able to explain it in detail.” (Participant 01, Hospital 03, Nurse practitioner) “Well, I would say, the patient has to believe in the benefits of the treatment, […] That’s it, they have to believe in it, they have to believe in it in order to stick with it.” (Participant 07, Hospital 01, Manager) “But I have the impression that our sales pitch is still worth something, in the sense of just encouraging them by telling them: ‘you know, actually, the results are good, the data shows it’s effective.’” (Participant 04, Hospital 02, Psychologist) “Even at one point, I spoke to a patient again, and it had changed their life. Of course, that kind of motivated me to keep offering it” (Participant 04, Hospital 01, Pharmacist)
Readiness	Patient	“That’s not as bad, because it’s big and quite visual and all that, but it’s the reading. I have severe glaucoma—I can see, but… well, it’s not going well.” (Participant 06, Hospital 02) “I think some patients might be more elderly and they’re not that comfortable with the Internet.”(Participant 02, Hospital 02) “So what also kept me in the program is the fact that it didn’t take up much of my time to fill in the questionnaires every day and, well, the summary of our night and all that.” (Participant 04, Hospital 01) “Well, that’s what I really liked, that you have one module per week, a person isn’t overloaded with information. You could take the time to absorb it and then to apply it in your daily life. So that was fantastic.” (Individual interview I001, Hospital 02)
	Provider	“Yes, I also think it’s about finding the right patient, and at the right time in their journey. You know, insomnia can also be related to many things, and it really depends on where they are in terms of diagnosis versus treatment, or, you know, if anything is happening in their personal life.” (Participant 02, Hospital 04, Nurse) “The people who are referred are also selected. Of course, an 85-year-old man with cognitive trouble might not be the target clientele, but I think part of it, like participant 1, is also the fact that when referring—you consider that it’s somebody who would be able to do the program and that they have a sufficient level of literacy. So, I don’t think it’s an issue for me in terms of comprehension. I think the patients who are referred generally understand well enough. The material is pretty simple, too.” (Participant 02, Hospital 03, Nurse navigator) “I noticed that for some patients, barriers had mostly to do with accessibility. For instance, if they don’t have a computer or tablet, then they have to use a smartphone, which doesn’t work as well with the platform. Also, depending on where somebody is in their care journey, I found that could be more challenging.” (Participant 01, Hospital 02, Psychologist) “Well, once you’re there, you quickly fall back into your old habits. So I often realize the fact that it doesn’t really come naturally, you know, to refer patients to the program.” (Participant 06, Hospital 02, Nurse navigator)
Perceived burden	Patient	“For me it came at just the right time, I was housebound, I think this was in 2021 so I was sort of able to devote myself to it. Sometimes I take a step back from the pace of the life we lead, because there are parts I find tough.” (Participant 03, Hospital 02) “Well, it helped me a lot, and then the fact that it’s online means that it was something I followed through with because you can do it any time you want.” (Participant, 01, Hospital 02)
	Provider	“So it fit into our schedules pretty well. In the end, it didn’t really become an extra burden. At first, there were…there were concerns—managers worried, staff worried, even the psychologists themselves—but in the end that didn’t happen. Pretty quickly, people were reassured that it didn’t actually take that much time.” (Participant 04, Hospital 02, Psychologist) “We still had good follow-up from the research team—they would remind us and give us tips. We felt supported throughout the whole process. It wasn’t like, ‘Okay, from now on you just do this on your own and we’re done.’ We really did have support from them.” (Participant 03, Hospital 02, Pharmarcist) “Yeah, in terms of time, our staff—sure, when we do an effective intervention and try to cover everything, we always run short on time. But as a team, that’s kind of the message we were giving within the group: if there’s a start to an intervention done for Participant 1 who’s being assessed, and then followed up by the nurse navigator, the psychologist will also complete it, the pharmacist… I think it’s really a group thing and not something that depends on one single person. So that’s the approach we were telling our staff: ‘Just get it started, even if it means the nurse navigator ends up calling the patient back to complete the information.” (Participant 03, Hospital 04, Manager)
Fit	Patient	“We chat a lot among ourselves in the waiting rooms. Some people don’t go through chemo, there are some who go straight to radiation—they don’t have any information at all. Having done both, I got zero information. I’d show up in the evening, everything closed, no receptionist, and someone would just grab me, put me in the machine, and say ‘thanks, goodnight.’ That’s why I think it really should be the nurse navigator or the doctors who take the time to explain things.” (Participant 04, Hospital 02) “For the program, they just gave me the little pamphlet. That was way better than explaining it to me, because when they explained, I was like, ‘Whoa, hold on a sec.’ Coming out of an appointment, you’re just not ready for that. Not mentally ready. So the little paper—that works.” (Individual interview, I001, Hospital 02)
	Provider	“Because, yes there were some meetings, but, well, I have to confess that I forget, even if I have the little bookmarks, everything is there, but I don’t think about it all the time. So maybe a little email reminder now and then, that could work and it’s personal.” (Participant 02, Hospital 03, Nurse navigator) “You can tell there’s not much engagement on the part of the patients, so we offer it, but we get turned down pretty quickly, and after a while we just do it less.” (Participant 01, Hospital 04, Nurse) “I don’t think we really have time to talk much with patients about the program. Except for a couple of lines, when we tell them it works as well as the medication, but anyway. In the real world, we just don’t have the resources to talk about the program for long.” (Participant 07, Hospital 02, Physician) “You know, medication is still the easy way, both for us and for the patient as well. So I think that’s one issue that worked against us from the team’s perspective.” (Participant 02, Hospital 04, Nurse) “As more new people come in who weren’t around for the development and rollout, and fewer and fewer of the people who were there, there’s a risk we’ll eventually lose the thread and no longer know, ‘What was this thing again?’” (Participant 04, Hospital 02, Psychologist)
Additional considerations	Patient	“What I think would help is talking to someone along the way after a couple of weeks, just to make adjustments.” (Individual interview, I004, Hospital 02) “I don’t really feel like going through all the readings again, but I’d like to remind myself of a bit of each, because that would help keep them fresh in my memory. Sometimes you want to do everything right in life, but there are things you forget to apply. Not because you don’t know that you should do them, but just because you forgot. And then you think, ‘Hey, I could do that—it’d be easy and it would help me.’ So a little reminder would be like a wake-up call.” (Participant 01, Hospital 02)
	Provider	“Well, that depends. Which organization is going to take responsibility for keeping a platform up to date, making sure it works, if there are any bugs, who handles that, who pays the IP people? Right now, this was all done by the project.” (Participant 01, Hospital 01, Manager) “The main thing is you need to make sure there’s someone responsible. And that there’s an update. You know, the software, the site—it’s great, it’s up to date now, but in ten years’ time it might be something else, so you definitely need someone to keep it alive. And if it’s someone external, what’s interesting is that this is a program that’s implemented not just here, but in other facilities in your region too. That way, you can be sure everyone has the same setup, and if there’s an update, everyone gets the same update, which is important. But if it’s someone internal, well, maybe then we can’t maintain that link with the other people.” (Participant 01, Hospital 03, Nurse practitioner)

## Data Availability

Data are available from the authors upon reasonable request.

## Supplementary Materials

The following supporting information can be downloaded at: https://www.mdpi.com/article/10.3390/curroncol33100588/s1, Supplementary Material S1: COREQ (COnsolidated criteria for REporting Qualitative research) Checklist [37].

## Abbreviations

The following abbreviations are used in this manuscript:

CBT-I	Cognitive-Behavioral Therapy—Insomnia
RCT	Randomized Controlled Trial
IMPACT	Insomnia in Patients with Cancer—Personalized Treatment
ESAS-R	Edmonton Symptom Assessment System—Revised
ISI	Insomnia Severity Index
CFIR	Consolidated Framework for Implementation Research

## Appendix A

A semi-structured interview guide based on the CFIR domains was designed. The English version is found in Table A1. The interview guide differed for clinicians and patients only in the overall assessment and recommendations sections. Within those sections, questions oriented to providers and clinicians were bolded.

**Table A1:** Semi-structured interview guide.

Theme	Questions
Overall assessment	Were you interested by the inclusion of this service in routine care? For what reasons (interest or lack of interest)? What did you find helpful in the implementation strategies that were used? What could have been done differently to help you benefit from the program? Overall, what did you think of the IMPACT program (the online program and the additional booster sessions)? Was everything clear? If not, what wasn’t clear? Was the program effective in reducing your trouble sleeping? If not, or if the effects were not sustained over time, what was missing from the program? Do you think that in the future patients who participate in the program should be given more support? For what reasons? For patients who were offered booster sessions at the end of the program but who didn’t receive them: What was/were the reason(s) why you did not request access to additional sessions with a psychologist when they were offered to you at the end of the program? (e.g., requested to have support for another issue, already receiving support for another issue, lack of time/interest in meeting a psychologist, sleep was satisfactory after completion of the IMPACT program) In your opinion what was the reason why a large number of the patients who were referred to the program (who were given a referral card) did not sign up? Could anything be done to increase the uptake rate, to make the program more attractive/easier for patients? **Overall, how is the IMPACT program being rolled out in your institution?** ▪ **ASK AS NEEDED: Worse or better than expected? As well or as poorly as expected?** **To what extent do you (or other providers) find it useful to be able to refer patients with insomnia symptoms to this program (scale from 1 to 10)?** ▪ **ASK AS NEEDED: Do they see benefits to including this program as part of routine care for patients? Are there any drawbacks? Does it address a need?** **Has adding the IMPACT program to routine care at your institution affected the overall quality of care? If yes, how?**
Patient-related barriers and facilitators	*Barriers* In your view what kind of patient-related barriers do patients face when they are referred to the IMPACT program? ▪ Was this program easy to put into practice in your daily life? Why or why not? ▪ ASK AS NEEDED: insomnia is not an important enough problem to sign up, lack of motivation, fatigue, not comfortable with the Internet, trouble understanding health information, a feeling that the program is too complicated, no time, would have liked to be treated by a professional, forgot, etc. ▪ In your view, what solutions were tried in order to overcome these obstacles? Did they work? ▪ What other solutions could be tried? *Facilitators* In your view, what factors for patients referred to IMPACT would make it easier for them to use the program? ▪ Was the program easy to put into practice in your daily life? ▪ ASK AS NEEDED: motivation, comfortable with Internet, complete the program at your own speed, without having to go to the hospital, interested in non-pharmacological approaches, desire to be actively involved in your own recovery
Intervention-related barriers and facilitators	*Barriers* In your opinion what barriers are related to the intervention itself that make it harder for patients to participate in IMPACT? ▪ ASK AS NEEDED: Complexity, user costs, security, need for an Internet connection and to be sufficiently computer literate, CBT requires major changes in personal habits, etc. ▪ In your view, what solutions were tried in order to overcome these obstacles? Did they work? ▪ What other solutions could be tried? *Facilitators* In your opinion what factors related to the intervention itself make it easier for patients to participate in IMPACT? ASK AS NEEDED: Accessibility (simple and suggested at the right time) access free of charge, long term beneficial effects for patients, etc.
Institution-related barriers and facilitators	*Barriers* In your opinion, what institution-related barriers do patients face while participating in IMPACT in clinics? ▪ ASK AS NEEDED: time constraints, workload and schedule, lack of communication and sharing of information, hospital regulations, restructuring of services (e.g., relocation of cancer centre), etc. ▪ In your view, what solutions were tried in order to overcome these obstacles? Did they work? ▪ What other solutions could be tried? *Facilitators* In your opinion, what institution-related factors make it easier for patients to participate in IMPACT in clinics? ASK AS NEEDED: hospital flexibility, culture and openness to change and to new methods, intervention that responds to a need or to a situation observed within the institution, little additional workload, communications, the centre’s willingness to provide more innovative care, presence of clinician advocates who promote the program, etc.
External barriers and facilitators	*Barriers* In your opinion, what barriers external to institutions make it harder for patients to participate in IMPACT? ▪ ASK AS NEEDED: COVID-19 pandemic (temporary suspension of paper documentation), offloading of clinical activities, redeployment of certain staff, etc. ▪ In your view, what solutions were tried in order to overcome these obstacles? Did they work? ▪ What other solutions could be tried? *Facilitators* In your opinion, what factors external to institutions make it easier for patients to participate in IMPACT? ASK AS NEEDED: COVID pandemic and increased use of digital health and email to communicate with patients.
Staff-related barriers and facilitators	*Barriers* In your opinion, what staff-related barriers do patients using IMPACT face? ▪ ASK AS NEEDED: Resistance to change, lack of interest in the problem (insomnia) or in the approach (non-pharmacological), lack of flexibility, of abilities, of confidence, etc. ▪ In your view, what solutions were tried in order to overcome these obstacles? Did they work? ▪ What other solutions could be tried? *Facilitators* In your opinion, what staff-related factors make it easier for patients to participate in IMPACT? ASK AS NEEDED: Flexibility, interest in the problem (insomnia) or in the approach (non-pharmacological), desire to provide better care for patients, feeling of competence and of confidence, motivation, etc.
Implementation durability	In your opinion, should the IMPACT program continue to be offered in routine care once the research project has concluded? Why? In your opinion, how likely is it that the IMPACT program will continue to be offered as part of routine care in cancer clinics to patients who have trouble sleeping, once the research project has concluded? For what reasons? Do you have any concerns or apprehensions about the long-term sustainability or continuation of the IMPACT program in routine care? If so, what are they? What recommendations do you have for the research team regarding the implementation of the IMPACT program in institutions? ▪ ASK AS NEEDED: adapt the intervention, make it more flexible and user friendly for patients, provide telephone support, etc. ▪ **ASK AS NEEDED: adapt the intervention to continue offering it in the long term (while maintaining its core components), identifying key individuals for maintaining the program in each institutions (clinician champions), addressing staff turnover, etc.** **Which implementation strategies did you find helpful (e.g., distribution of promotional material to providers, holding quarterly meetings with providers to review the project, present statistics, answer questions, distribution of simple material related to the project)? What else could have been done to help you refer patients to Insomnet?** **Do you think the program will continue to be offered to patients who have sleep difficulties in your institution (by you and/or by all other providers involved) once the research project is over from May 2024? For what reasons?** **If at some point Insomnet were to be offered by a third party (e.g., a community organization’s platform), would you still be inclined to refer patients to it? Why or why not?** **Does the intervention contribute to inequality in healthcare (e.g., for people without a computer, with low literacy, or who do not speak French)? How can we make it more accessible and keep it fair for different groups and clinical settings (DEI)?**
Conclusion	Do you have any questions or comments about the sustainment of the implementation of the IMPACT program in oncology clinics?
